# Effect of multidisciplinary health education based on lifestyle medicine on menopausal syndrome and lifestyle behaviors of menopausal women: A clinical controlled study

**DOI:** 10.3389/fpubh.2023.1119352

**Published:** 2023-03-16

**Authors:** Yangmei Li, Haiyang He, Jiaxi Wang, Yifan Chen, Chunyuan Wang, Xinyue Li, Anqi Dai, Yue Liu, Xin Xi, Juan Huang, Mi Zou, Yao Fan, Mingfang Zhou, Ping Yi, Lili Yu, Xun Lei

**Affiliations:** ^1^School of Public Health, Chongqing Medical University, Chongqing, China; ^2^Research Center for Medicine and Social Development, Chongqing, China; ^3^Collaborative Innovation Center of Social Risks Governance in Health, Chongqing Medical University, Chongqing, China; ^4^Research Center for Public Health Security, Chongqing Medical University, Chongqing, China; ^5^Department of Obstetrics and Gynecology, The Third Affiliated Hospital of Chongqing Medical University, Chongqing, China

**Keywords:** menopausal women, multidisciplinary, health education, lifestyle medicine, menopausal hormone therapy

## Abstract

**Background:**

Menopausal women may experience menopausal syndrome and long-term effects caused by low estrogen levels, such as senile dementia and osteoporosis in the elderly. Most menopausal women may have misconceptions about menopause and low use of pharmacological interventions. These misconceptions may damage the quality of life and miss the critical period for preventing senile diseases. Thus, enhancing the awareness of menopausal women regarding psychosocial and physical changes through health education programs was a way to improve positive attitudes toward menopause and make further treatment options.

**Objectives:**

This study aimed to evaluate the effect of multidisciplinary health education based on lifestyle medicine on menopausal syndrome and lifestyle behaviors of menopausal women.

**Methods:**

The study was conducted in several hospitals in Chongqing, China. The two groups were from different hospitals with similar medical levels in order to reduce information contamination. It was designed as a clinical controlled trial in which the intervention group (*n* = 100) and control group (*n* = 87) were matched for age, age at menarche, menopausal symptoms and drug use status at enrollment. Women in the intervention group received multidisciplinary health education based on lifestyle medicine for 2 months while those in the control group received routine outpatient health guidance. Menopausal syndrome, physical activity and dietary status of participants were assessed before and after the intervention. Paired *t*-tests and Independent-sample *t*-tests were adopted for comparison within and between groups, respectively, in the normal variables. Wilcoxon signed-rank tests and Mann-Whitney U tests were adopted for comparison within and between group, respectively, in the abnormal variables. Categorical variables were tested using Pearson's χ^2^. *P*-value < 0.05 was statistically significant in statistical tests.

**Results:**

Post intervention testing indicated that menopausal syndrome of participants was significantly improved in the intervention group compared to the control group (*P* < 0.001). Between-group comparison showed a significant improvement of weekly energy expenditure of total physical activity (*P* = 0.001) and participation in exercise (*P* < 0.001) in the intervention group compared to the control group after the intervention. The dietary status of participants was significantly improved in the intervention group compared to the control group (*P* < 0.001). In the intervention group, the menopausal syndrome of participants improved more in the hormone drug group than in the non-hormone group (*P* = 0.007), as did the control group (*P* = 0.02). In the hormone drug group, the physical activity (*P* = 0.003) and dietary status (*P* = 0.001) mproved more in the intervention group than in the control group.

**Conclusions:**

The multidisciplinary health education based on lifestyle medicine was effective in improving the menopausal syndrome and healthy lifestyle behaviors of menopausal women. Studies with extended observation period and larger sample size are in need to evaluate the long-term scale-up effects of the multidisciplinary health education.

## 1. Introduction

Women typically enter menopause gradually at the age of 40 and continue until the age of 60. Due to declining ovarian function and falling estrogen levels, women in this period may experience menopausal syndrome which may manifest as hot flashes, arthralgia, insomnia, depression and so on (1). Long-term effects caused by low estrogen levels, such as senile dementia and osteoporosis, may seriously affect health and life quality in the older women. Although life expectancy has gradually increased over the last 100 years due to demographic shifts, the age at menopause has not changed. For this reason, women may spend several decades of their lives in the postmenopausal period. Menopause is an important women's health issue to be considered in the planning and presentation of health services (2).

Despite many complaints that menopausal women may experience, there are some medical measures that can be improved and prevented. Menopausal hormone therapy (MHT) may be one the most effective measure to alleviate women's menopausal syndrome and prevent senile dementia and osteoporosis in the elderly (3). There are some guidelines and expert consensus around the world that recommend it for women with no contraindications in early menopause (3–6), however, there may be minor side effects of MHT, such as an increased risk of breast cancer and cardiovascular events (4). It is important for women to have a thorough understanding of MHT and benefits and risks in order to use it.

Despite the acknowledged benefits of MHT, many menopausal women have misconceptions about menopause and low use of pharmacological interventions, which may depend on cultural, social and lifestyle factors (7–10). These misconceptions may damage the quality of life and miss the critical period for preventing senile diseases. Enhancing the awareness of menopausal women regarding psychosocial and physical changes through health education programs is a way to improve positive attitudes toward menopause and make further treatment options (11). The studies (7, 12–14) have suggested that healthcare providers should create more in-depth education programs so that women can seek advice on how to optimally manage their menopause and the years beyond.

Lifestyle Medicine (LM) is a growing field of medicine, which is most comprehensively defined as “the integration of lifestyle practices into the modern practice of medicine both to lower the risk factors for chronic disease and/or, if disease already present, serve as an adjunct in its therapy” (15). Lifestyle medicine is a rapidly evolving field that addresses the lifestyle-related contributors to non-communicable diseases by changing health behaviors. In the guideline (3) for menopausal women, lifestyle medicine is proposed as the basic treatment for women with menopausal syndrome. Unhealthy lifestyles such as improper dietary practices and lack of physical activities aggravated the consequences of estrogen level changes (16). Moreover, some studies (17, 18) have shown that a healthy lifestyle can reduce or even counteract the risks of MHT. Thus, lifestyle medicine can also be used in menopausal women' health management. Lifestyle medicine need references for the multiple study populations such as hypertension, diabetes and polycystic ovary syndrome patients.

Physical exercise and healthy diet have many benefits for menopausal women. Exercise has been assessed as an alternative treatment option for alleviating menopausal symptoms, including, psychological, vasomotor, somatic and sexual symptoms in Stojanovska et al.'s study (19). A higher level of physical activity during the different menopausal phases was beneficial, especially for skeletal muscle (20). A healthy diet can improve women's health problems such as obesity and metabolic syndrome (21).

Even though previous studies (22, 23) have shown positive effects of health education on menopausal women, most of them were single-subject education, such as teaching only about menopause or healthy life education. Pelit Aksu and Sentürk Erenel's study has shown that health education and progressive muscle relaxation were effective in managing vasomotor symptoms and insomnia (24). Another study by Mirhosseini also demonstrated the positive role of an online psychoeducational support group in perceived stress (25). Moreover, a study has shown the positive effect of trans-theoretical model-based health education program on the management of cognitive dysfunction in older adults (26). Due to the particularity of menopausal women in complex symptoms, psychological characteristics, comprehensive interventions from a multidisciplinary, multimodal approach should be implemented (27). Therefore, this study was designed to evaluate the effectiveness of a multidisciplinary health education based on lifestyle medicine (MHELM) on menopausal syndrome and lifestyle behaviors in menopausal women.

## 2. Methods

### 2.1. Study setting and participants

The study was conducted in six hospitals in Chongqing, China from August 2021 to August 2022. Participants were from different hospitals with Grade-A Tertiary Hospital in order to reduce information contamination. Located in southwestern China, Chongqing is known as a “miniature of China” because its urban-rural distribution, social-economic profile, and geographic characteristics were close to the national average. Currently, Chongqing consists of 38 districts and counties with 34.13 million residents, among which middle-aged woman account for 38.7% (28).

Participants were recruited in the study if they met the following inclusion criteria: women aged 40–60 years and suffered from menopausal syndrome assessed by the modified Kupperman Menopause Index (mKMI). The following exclusion criteria were applied: (1) without these diseases such as malignant tumors/severe organic dysfunction/acute infectious diseases/severe osteoporosis; (2) receiving psychological treatment; (3) cannot exercise.

### 2.2. Sample size calculation

Sample size for the study was decided by comparing the KMI before and after the MHELM among participants in polit, using the formula $n_{1}=n_{2}=2{\left[\frac{\left(z_{\alpha /2}+z_{\beta }\right)\sigma }{\delta }\right]}^{2}$. The value of σ^2^ was 52.37 and the value of δ was 13.76. Meanwhile, the value of α was 0.05, and the value of β was 0.20. Allowing for 15% of dropouts, the minimum required sample size was 72 for each group.

### 2.3. Intervention

**Figure 1:**
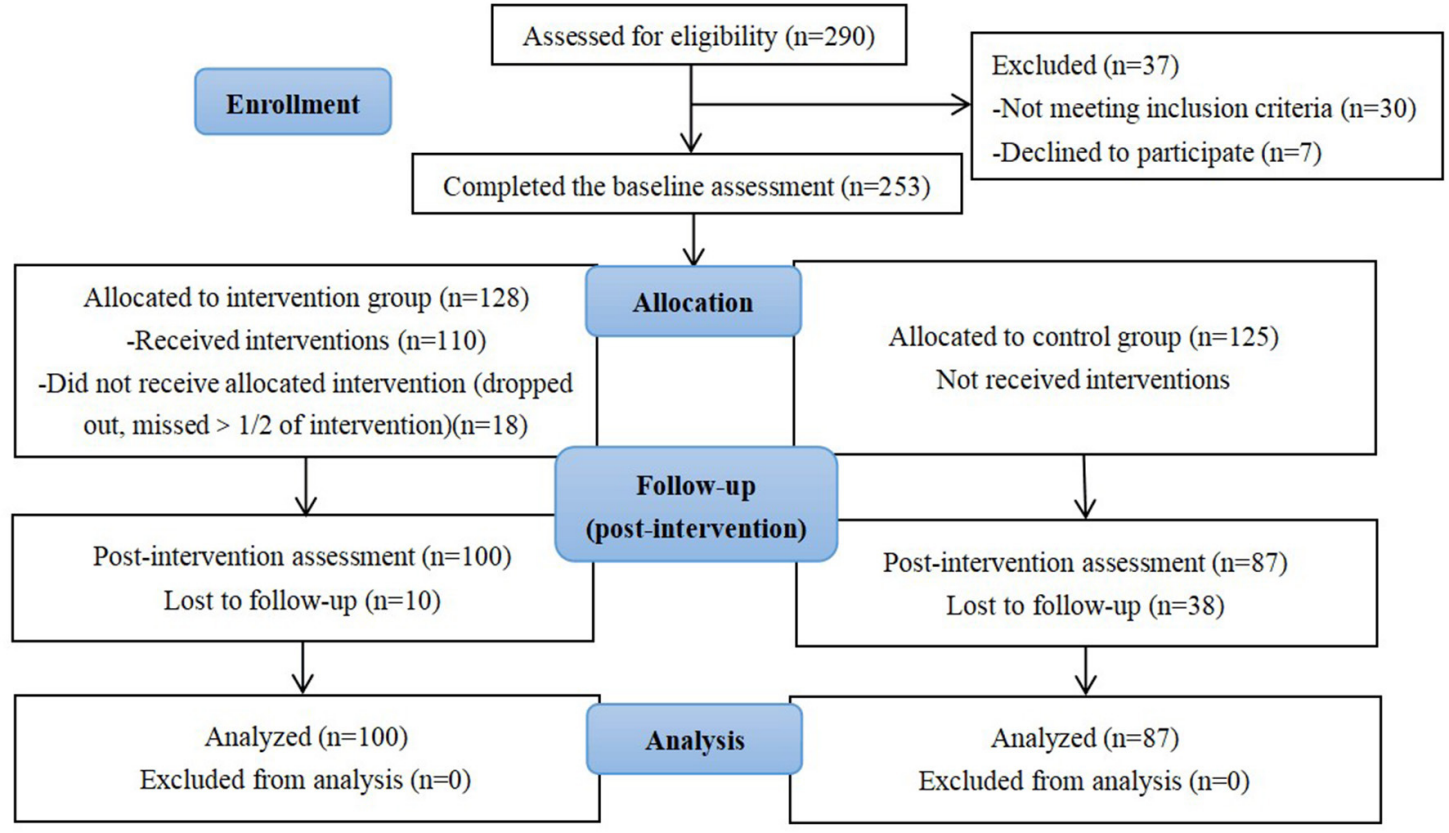
CONSORT flow chart.

This study was designed as a clinical controlled trial in which intervention group and control group were matched for age, age at menarche, menopausal symptoms and drug use status at enrollment. The criterion of matching referred that no statistical significance was detected in the difference analyses of baseline indicators according to clinical consideration (Table 1). After enrollment, the intervention group received MHELM while the control group received routine outpatient health guidance. Socio-demographic characteristics, signs of menopausal syndrome, physical activities, and dietary status were interviewed at the beginning and end of the study. Posttests were conducted with the participants in two groups immediately at the end of the 2-month period of the study. The study flowchart was shown in Figure 1.

**Table 1:** Comparison of the baseline characteristics between groups.

	**Intervention group**	**Control group**	***t/z/χ^2^*-value**	***P*** **-value**
	**(*n* = 100)**	**(*n* = 87)**		
Age (year)	47.55 ± 5.15	47.95 ± 4.11	−0.60	0.55
Age at menarche (year)	13 (12, 14)	13 (12, 14)	−0.80	0.43
Level of education, *n* (%)			3.31	0.19
Bachelor degree or above	26 (26.0%)	27 (31.0%)		
Senior Secondary and Junior College	51 (51.0%)	33 (37.9%)		
Primary and below	23 (23.0%)	27 (31.0%)		
Personal monthly income (RMB), *n* (%)			7.60	0.02
≥10,000	13 (13.0%)	3 (3.4%)		
5,000–9,999	23 (23.0%)	31 (35.6%)		
< 5,000	64 (64.0%)	53 (60.9%)		
Reproductive number, *n* (%)			5.73	0.02
Once or less	80 (80.0%)	56 (64.4%)		
Twice or more	20 (20.0%)	31 (35.6%)		
Severity of menopausal symptoms (mKMI score)	15.23 ± 6.49	17.36 ± 8.56	−1.89	0.06
Drug use status			1.11	0.29
Hormone drug	56 (56.0%)	42 (48.3%)		
Non-hormone drug	44 (44.0%)	45 (51.7%)		

Two group were matched for age, age at menarche, menopausal symptoms and drug use status.

#### 2.3.1. Multidisciplinary health education based on lifestyle medicine

MHELM comprised of three parts: 1-day Multidisciplinary Health Education Sessions offline, 7-week Group Healthy Lifestyle Management online, and half-day Patient Symposium offline.

##### 2.3.1.1. One-day multidisciplinary health education sessions

The sessions were provided by a multidisciplinary team (MDT) consisting of six experts under the professional background of gynecologic endocrinology, sports medicine, nutrition, music therapy, psychology, pharmacy, and osteoporosis. The sessions focused on menopausal symptom management, encouragement of physical activity, healthy diet, and spiritual support, osteoporosis prevention, pelvic muscle rehabilitation and medication guidance. The key topics of the sessions included the importance of menopause, the health implications of hypoestrogenism, the risks and benefits of hormonal and complementary therapy for menopausal symptoms and the importance of lifestyle medicine in the menopause stage, particularly on important topics of nutritionally-balanced diets and exercise (Table 2). Each episode of the 1-day multidisciplinary health education sessions, targeting 15–20 menopausal women, were held in the hospital every 2 months.

**Table 2:** The 1 day of multidisciplinary health education sessions flow.

**Schedule**	**Sessions**
8:00–9:20 a.m.	“Proper nutrition and diet management for perimenopausal women (interpretation of body composition reports)” and answers to questions – clinical dietitians
9:30–10:30 a.m.	“Rational drug use and guidance for perimenopausal women”—pharmacist
10:30–12:00 a.m.	“Scientific physical exercises and psychological regulation for perimenopausal women”—sports instructor and psychological consultant
12:00–13:00 p.m.	Experience nutritious meals
14:00–16:00 p.m.	“Coping with menopause – correct use of menopausal hormone therapy” (interpretation of female hormone test report) and answering questions-perimenopausal clinic physicians
16:10–16:50 p.m.	“Prevention and treatment of osteoporosis in perimenopausal women” and answers to questions—osteoporosis clinic physicians
17:00–17:40 p.m.	“Perimenopausal women's pelvic floor health” and answers to questions—pelvic floor center rehabilitation physiotherapist
17:40–18:00 p.m.	Organize medical records

##### 2.3.1.2. Seven-week group healthy lifestyle management

Seven-week Group Healthy Lifestyle Management following the 1-day multidisciplinary health education sessions were provided by professionals through WeChat. The content of the online group intervention included: reading, audio and video products of science popularization on the knowledge of menopausal health, reasonable diet and exercise. Psychological counseling was also provided based on music therapy, including music meditation, music rhythm, painting with music, music singing and listening to pure music. WeChat log of the daily physical activities and diet of participants were shared to professionals for assessment and guidance. Additionally, reminders were given by phone calls to the women who failed to adhere.

##### 2.3.1.3. Half-day patient symposium

After completing the 7-week Group Healthy Lifestyle Management, patients revisited the hospital for the Half-day Patient Symposium. In the symposium, experts reemphasized the importance of MHELM, and participants were encouraged to share their feelings and experience on MHELM. Participants who completed the whole process of MHELM would obtain a small gift.

#### 2.3.2. Control group

Participants in the control group filled out the pre- and post- questionnaires with a 2-month interval of follow-up, and in between they only received routine outpatient health guidance from perimenopausal physicians on the knowledge of perimenopausal symptoms and healthy lifestyle at enrollment.

#### 2.3.3. Ethical considerations

All the participants voluntarily signed the informed consent before the study. This study was approved by Institutional Review Board of the Third Affiliated Hospital of Chongqing Medical University (Number: 202112) and has been registered in Chinese Clinical Trial Registry (Registration number: ChiCTR2100049969).

### 2.4. Outcomes measurement

All participants filled out a pre-testing questionnaire at baseline and a post-testing questionnaire after 2 months. The primary outcomes included changes of women's menopausal syndrome and lifestyle behaviors such as physical activity and dietary status. Participants' feedback of MHELM group were interviewed as the second outcome for qualitative summary. The Questionnaires consisted of modified Kupperman Menopausal Index (mKMI) for menopausal syndrome, International Physical Activity Questionnaire (IPAQ) for physical activities and Dietary Habits Questionnaire. Socio-demographics regarding age, age at menarche, level of education, income, reproductive number and drug use status were collected as well.

#### 2.4.1. mKMI for menopausal syndrome

Menopausal syndrome was evaluated by the mKMI, which was a widely used scale to measure the presence and severity of menopausal syndrome. mKMI consisted of 13 items assessing the situation of women over the past week. The items included somatic, psychological and urogenital symptoms. The severity of each symptom was rated on a scale ranging from 0 to 3 (0, none; 1, mild; 2, moderate; 3, severe). The total scores ranged from 0 to 63; the higher the scores were, the more severe the symptoms would be. Severity of menopausal syndrome was categorized as: none (0–5 points); mild (6–14 points); moderate (15–30 points); severe (>30 points) (29).

#### 2.4.2. IPAQ for physical activities

IPAQ long form was applied to assess physical activity (PA) of the participants. It consisted of 27 items which reflected on the consumed time on walking and engaging in moderate-intensity plus vigorous-intensity activities in terms of different domains in the past week: (1) occupational PA; (2) traffic PA; (3) housework PA; (4) exercise PA; and (5) time spent sitting. Occupational, traffic and housework PA can be integrated as non-exercise daily PA. Weekly energy expenditure of total PA, non-exercise daily PA and exercise PA (MET-min week^−1^) were estimated by adding the sections of reported time for each item by a MET value that was specific to each category of PA (walking = 3.3 METs, moderate-intensity PA = 4.0 METs and vigorous-intensity PA = 8.0 METs) (30).

#### 2.4.3. Dietary habits questionnaire

Dietary status of the participants was assessed by the Dietary Habits Questionnaire, which was adapted from a questionnaire developed by the National High-tech Health Industry Working Committee of China and took account of dietary characteristics of menopausal women (31). It consisted of 30 questions assessing food intake and dietary habits of participants. The total scores were the summation of each item ranging from 1 to 4 scores; the higher the scores were, the worse the dietary status would be.

#### 2.4.4. Participants' feedback in the intervention group

Feedback from the participants in MHELM, including feelings, experience and suggestions, were collected through focus group discussions (FGDs) during the Half-day Patient Symposium. The process of discussions were assessed by perimenopausal physicians and recorded by two other professionals. Thematic analysis method was used to summarize the highlighting viewpoints.

### 2.5. Statistical analysis

Continuous variables that were normally distributed were presented with mean ± standard deviation, and compared using Paired *t*-tests and Independent-sample *t*-tests for statistical difference within and between groups, respectively. Continuous variables that were abnormally distributed were presented with medians (25th percentile, 75th percentile), and compared using Wilcoxon signed-rank tests and Mann-Whitney *U*-tests for statistical difference within and between groups, respectively. Categorical variables were presented by number (percentage) and tested using Pearson's χ^2^. Two-sided *P*-value < 0.05 was statistically significant. All data analyses were performed using the IBM SPSS (version 23.0, Armonk, NY).

## 3. Results

### 3.1. Sociodemographics and baseline characteristics

Table 1 showed that personal monthly income and reproductive numbers were statistically different (*P* < 0.05) between intervention and control groups at baseline. There were no significant differences in the baseline data such as age, age at menarche, level of education, and severity of menopausal symptom between the two groups. Drug use status of participants were also not statistically different between the two groups and remain unchanged during the study period.

### 3.2. Comparison of women's menopausal syndrome, lifestyle behavior

Comparisons of mKMI, IPAQ and dietary status within and between groups were listed in Table 3. The scores of mKMI assessing menopausal syndrome in both groups were significant lower in the posttest compared to the pretest (*P* < 0.001). No significant difference was detected for mKMI scores between two groups in the pretest (*P* > 0.05), however, scores of the intervention group was significantly lower than that of the control group in the posttest (*P* < 0.001). Pre- and post- testing showed that the total weekly energy expenditure of PA and weekly energy expenditure of exercise PA increased significantly in the intervention group during the study period (*P* < 0.01), while it was not significantly different in the control group (*P* > 0.05). Between-group comparisons showed a significant improvement of total weekly energy expenditure of PA and weekly energy expenditure of exercise PA in the intervention group compared to the control group in the posttests. In addition, the score of dietary status decreased significantly in the intervention group during the study period (*P* < 0.001), while no significant difference was detected in the control group (*P* > 0.05). Between-group comparisons showed a significant improvement of dietary status in the intervention group compared to the control group in the posttests.

**Table 3:** Comparison of mKMI, IPAQ and dietary status within and between groups.

		**Pretest**	**Posttest**	***t/z*** **-value**	***P*** **-value**
Menopausal syndrome	Intervention group	15.23 ± 6.49	9.88 ± 6.38	−7.55	<0.001
	Control group	17.36 ± 8.56	13.75 ± 8.00	−3.66	<0.001
	*t/z*-value	−1.89	−3.67		
	*P*-value	0.06	<0.001		
Total weekly energy expenditure of PA	Intervention group	2,143.5 (1,202, 4,031)	2,686.5 (1,179, 5,764)	−2.72	0.007
	Control group	2,079.5 (1,155, 2,780)	1,965.5 (495, 3,402)	−1.86	0.06
	*t/z*-value	−0.79	−3.42		
	*P*-value	0.432	0.001		
Weekly energy expenditure of non-exercise PA	Intervention group	1,393 (640.5, 2,709)	1,171.5 (619, 3,190.5)	−0.96	0.34
	Control group	1,323 (495, 2,767.5)	1,022 (328.5, 2,065.5)	−2.36	0.02
	*t/z*-value	−0.63	−2.18		
	*P*-value	0.53	0.03		
Weekly energy expenditure of exercise PA	Intervention group	650 (198, 1,386)	1,386 (404, 2,573)	−4.30	<0.001
	Control group	660 (0, 1,386)	495 (0, 1,155)	−0.41	0.68
	*t/z*-value	−0.77	−3.92		
	*P*-value	0.44	<0.001		
Dietary status	Intervention group	55.54 ± 8.59	51.41 ± 6.81	−5.78	<0.001
	Control group	57.18 ± 10.00	59.21 ± 10.13	1.89	0.14
	*t/z*-value	−1.20	−6.25		
	*P*-value	0.23	<0.001		

PA, physical activity.

### 3.3. Changes in women's menopausal syndrome, lifestyle behavior according different drug use status

Comparisons of the changes on mKMI, IPAQ and dietary status were listed in Table 4. Both in the intervention and control groups, the scores of mKMI decreased more in the hormone drug group than in the non-hormone group (*P* < 0.05). In the hormone drug group, the changes of physical activities were significantly different between intervention and control groups (*P* < 0.05), with an increase in the intervention group and a decrease in the control group. In terms of dietary status improvement, there were significant differences between the intervention and control groups in both hormone and non-hormone drug groups (*P* = 0.001).

**Table 4:** Changes of KMI, IPAQ and dietary status within and between groups according different drug use status.

		**Hormone drug group**	**Non-hormone drug group**	***t*** **-value**	***P*** **-value**
Menopausal syndrome	Intervention group	−7.04 ± 6.41	−3.20 ± 7.40	−2.77	0.007
	Control group	−5.90 ± 9.41	−1.47 ± 8.55	−2.30	0.02
	*t/z*-value	−0.67	−0.65		
	*P*-value	0.51	0.31		
Total energy expenditure of PA	Intervention group	529.50 (−479, 2,002)	238.50 (−994, 2,203)	−0.80	0.42
	Control group	−584.75 (2,517, 10,140)	−288 (−1,267, 858)	−0.86	0.39
	*t/z*-value	−2.95	−1.56		
	*P*-value	0.003	0.12		
Energy expenditure of non-exercise PA	Intervention group	170.5 (−426, 907.5)	−13.75 (−858, 604.5)	−1.08	0.28
	Control group	−509 (−1,468.5, 544.5)	−219 (−945, 745)	−0.69	0.49
	*t/z*-value	−2.33	−0.94		
	*P*-value	0.02	0.35		
Energy expenditure of exercise PA	Intervention group	429 (−81, 1,414)	226 (−336, 1,688)	−0.60	0.56
	Control group	−33 (−1,070, 396)	0 (−308, 480)	−0.74	0.46
	*t/z*-value	−2.94	−1.71		
	*P*-value	0.003	0.09		
Dietary status	Intervention group	−4.00 (−8.75, 0.00)	−3.50 (−9.00, 1.75)	−0.31	0.76
	Control group	−1.00 (−4.00, 9.00)	4.00 (−4.00, 6.50)	−0.35	0.73
	*t/z*-value	−3.20	−3.43		
	*P*-value	0.001	0.001		

The value of comparison within and between groups is the change value of the test (the post-test value minus the pre-test value); PA, physical activity.

### 3.4. The participants' feedback in the intervention group

The women's feedback after participating MHPEI were summarized into the following four themes including relief of menopausal symptoms, awareness and behavior of menopausal health management, relief of negative emotions and improvement of menopausal cognition. Highlighting statements were shown in Table 5.

**Table 5:** Participants' feedback in the intervention group (60/100 = 60%).

1. Menopausal syndromes such as hot flashes, sweating, insomnia, and bone and joint pain were significantly improved in participants.
2. Strengthening the awareness and behavior of health management, such as enhancing the awareness of preventing chronic diseases and developing a healthy lifestyle.
3. The negative emotions such as irritability and depression were gradually improved.
4. Participants reported that it cleared up the misunderstanding of menopause cognition and increased their confidence in coping with menopause.

## 4. Discussion

The present study took the lead in exploring the effects of multidisciplinary health education intervention based on lifestyle medicine on menopausal women in China. The current study revealed that MHELM helped the women to feel relief from troublesome menopausal syndrome, improve healthy lifestyle behaviors and enhance awareness of menopausal health management.

In our findings, the menopausal syndrome of participants was significantly improved in the intervention group compared to the control group when drug usage was matched between groups. This confirmed that the MHELM intervention was effective in improving the menopausal syndrome. Our observations were consistent with some studies which reported the effectiveness of health education interventions (32–34). A Sri Lankan study (34) showed that health-promoting lifestyle education relieved menopausal symptoms and enhanced physical functions. Another study by Sehhatie Shafaie et al. (33) also demonstrated that education *via* support group reduced the distressing menopausal symptoms. However, inconsistent findings were reported in previous researches. The Rindner et al.'s study (35) did not show that group education intervention could reduce the somatic, urogenital and psychological symptoms of menopause. In Beylikova's intervention study (22), health education did not improve urogenital subtype symptoms of menopausal women. Therefore, single use of health education might be limited in improving menopausal symptoms, and multidisciplinary management based on MHT should be adopted when necessary.

We observed that the control group had a significant improvement on the symptoms of menopausal syndrome without MHELM intervention. Moreover, the results indicated that the menopausal syndrome of participants had a significant improvement in the hormone drug group compared to the non-hormone drug group. The evidence further confirmed that appropriate medical treatment, especially for MHT, should be taken to relieve the painful symptoms of menopausal women. Furthermore, the symptoms were essentially caused by estrogen deficiency in which case MHT could be the recommended measure to relieve them. Worldwide guidelines have allowed healthcare professionals to gain more clarity into the role of MHT, not only in the relief of troublesome menopausal symptoms, but also in the prevention of chronic diseases associated with aging (3–6). It was worth noting that MHT should be considered to be part of an overall strategy including health lifestyle recommendations regarding diet and exercise for maintaining the health of women.

In our study, the MHELM intervention has significantly increased the energy expenditure of physical activities, especially in exercise physical activities, and improved the dietary status of women compared to those in control group. Meanwhile, similarly significant improvements were observed in the hormone drug group. Although our findings were consistent with previous studies which reported the effectiveness of lifestyle interventions, previous studies on lifestyle interventions were implemented mostly from the perspective of individuals (23, 36). In contrast, MHELM intervention provided targeting subjects with continuous monitor and motivation on women's healthy diet and scientific exercise through a 7-week group healthy lifestyle management. In MHELM group, prolonged lifestyle intervention was highly effective in improving adherence and acceptability of participants. Furthermore, the basic measures of healthy lifestyle to prevent chronic diseases were also well-described in the theory of lifestyle medicine (37). This reaffirmed that our MDT intervention based on lifestyle medicine was extremely suitable for menopausal women.

Access to the correct information about menopause can help women have more realistic expectations about this period and make better choices among treatment options for coping with menopausal symptoms (38). However, previous studies reported that the majority of women lacked knowledge of menopause, denied MHT and were unwilling to seek medical advice (7–10, 39, 40). In health education for menopausal women, the primary aim was to help them to be familiar with this period, to eliminate inherent cognitive misunderstandings, and to positively change the way menopausal symptoms are perceived (23, 41). Our MHELM intervention adequately achieved this goal. One-day multidisciplinary health education sessions provided systematic and comprehensive knowledge on the nature of menopause and MHT treatment to the related health problems in this period, allowing women to have an initial understanding of menopause management. During the following 7 weeks of online WeChat group intervention, healthcare professionals continued to send participants relevant theories to further enhance their cognition and help them to make appropriate medical decisions. Finally, the participants returned to the hospital for a half-day symposium, by which the perimenopausal physicians reemphasized the importance of multidisciplinary management and provided consultation in detail. Moreover, in our study we conducted qualitative analyses on FGDs to avoid the limitations of questionnaires, which was the prospects of previous studies (34).

The effectiveness of the MHELM intervention depended on the MDT health management, useful lifestyle medicine theoretical framework and close progress monitoring. Thus, the success of our health education intervention would be attributed to a few reasons. Firstly, MHELM intervention was delivered by MDT from expertise of gynecologic endocrinology, sports medicine, music therapy, psychology, nutrition, pharmacy, osteoporosis. The core concept of MDT was patient-centered, individualized and continuously comprehensive treatment schemes for specific diseases based on the multidisciplinary team, which has been widely used over the world in recent years (42). Meanwhile, we innovatively combined music therapy in MHELM, which can relieve depression and menopausal symptoms to a large extent (43). Secondly, health education sessions were culturally accepted, multifaceted and well-designed program based on lifestyle medicine theoretical framework. The content validity of the program we developed was ensured by a group of experts. Thirdly, it also ensured a regular supervision in a 2-month follow-up, during which participants were motivated to learn the educational materials effectively and give timely feedback to professionals. Moreover, group interventions on lifestyle improved adherence of participants, meanwhile, mutual discussions in the group also had peer motivations and declined dropouts.

There were also several limitations of this study. Firstly, we only evaluated the short-term effects of MHELM and long-term effects need to be assessed by extended study period with a scale-up sample. Secondly, in our study only subjective indicators were involved in the assessment from self-reports by questionnaire, suggesting that clinical indicators such as body composition, blood lipid and glucose could be taken in account in future studies.

## 5. Conclusion

This study showed that multidisciplinary health education based on lifestyle medicine was effective in improving the menopausal syndrome and healthy lifestyle behaviors of menopausal women. The MHELM may lighten the design and implementation of similar interventions in future studies. Studies with extended Observation period and larger sample size are in need to evaluate the long-term scale-up effects of the multidisciplinary health education.

## Data availability statement

The raw data supporting the conclusions of this article will be made available by the authors, without undue reservation.

## Ethics statement

The studies involving human participants were reviewed and approved by the Institutional Review Board of the Third Affiliated Hospital of Chongqing Medical University (202112). The patients/participants provided their written informed consent to participate in this study.

## Author contributions

YML participated in field investigation, data collection, and drafting of the manuscript. HYH participated in the design of the study and field investigation and data collection. JXW, YFC, CYW, XYL, and AQD participated in field investigation and data collection. YL participated data analysis. YF, XX, JH, MZ, MFZ, and PY participated in the design of the study. XL and LLY participated in the design of the study, field investigation, data collection, and review of the manuscript. All authors saw and approved the final version.
